# Machine learning approaches for enhanced estimation of reference evapotranspiration (ETo): a comparative evaluation

**DOI:** 10.1038/s41598-025-23166-w

**Published:** 2025-11-04

**Authors:** Abousrie A. Farag

**Affiliations:** Department of Agricultural and Biosystems Engineering, Faculty of Agriculture, Benha University, Banha, Egypt

**Keywords:** Evapotranspiration, Machine learning, Random forest, K-Nearest neighbors, Decision tree, Irrigation management, Data processing, Machine learning

## Abstract

Accurate estimation of reference evapotranspiration (ETo) is critical for effective water resource management, particularly in regions with limited meteorological data. However, existing empirical and deep learning models often require extensive data or complex modeling, limiting their practical application in data-scarce environments. This study innovatively applies static (non-sequential) machine learning models K-Nearest Neighbors (KNN), Decision Tree (DT), and Random Forest (RF) justified by temporal dependency analysis, to estimate daily ETo using varying input scenarios, from full-feature datasets to minimal single-variable inputs. Results show that RF outperforms other models, achieving a root mean square error (RMSE) of 0.52 mm/day and a coefficient of determination (R²) of 0.96, with temperature and solar radiation identified as key predictors. These findings highlight the practicality of RF for robust and efficient ETo estimation, offering a reliable tool for water management and agricultural planning in resource-constrained settings.

## Introduction

 Agriculture is the largest global consumer of freshwater, accounting for nearly 70% of total withdrawals worldwide^1–3^. Inefficient irrigation practices and difficulty in accurately estimating crop water requirements often result in over- or under-watering, leading to water waste, plant stress, and reduced yields. These challenges are particularly acute in arid and semi-arid regions, where water scarcity threatens food security and economic stability^4–6^. Climate change exacerbates this situation by intensifying droughts, altering precipitation patterns, and increasing uncertainty in water availability, emphasizing the need for accurate water management strategies^7^.

Evapotranspiration (ETo), the combined loss of water through soil evaporation and plant transpiration—is a key factor in irrigation scheduling and water resource planning^8–10^. Numerous empirical and physically based approaches have been developed to estimate ETo using climatic variables such as temperature, solar radiation, relative humidity, and wind speed^11,12^. Among these, the FAO Penman–Monteith (PM) equation is widely considered the most reliable standard, as it incorporates multiple meteorological parameters^13^. However, the PM method requires extensive instrumentation, making it costly and impractical for regions with limited resources. In contrast, simpler equations, such as Blaney–Criddle, Priestley–Taylor, and Hargreaves–Samani, reduce data requirements but often compromise accuracy^14–16^. Direct measurement techniques, including lysimeters, Bowen ratio systems, and eddy covariance methods, provide localized precision but are resource-intensive and unsuitable for large-scale applications ^17^.

The cost of acquiring and maintaining sensors to measure climate parameters remains a major barrier to widespread implementation of high-accuracy models^18,19^. This issue is further magnified in large-scale monitoring networks or long-term studies, where the cumulative expense of high-precision equipment becomes prohibitive^15,16^. These challenges highlight the need for cost-effective, accurate approaches that reduce reliance on expensive instrumentation while maintaining robust performance.

Recent advances in machine learning (ML) have demonstrated considerable promise in modeling complex environmental processes, offering flexible and data-driven solutions without strict assumptions about system dynamics^20,21^. ML-based approaches have been successfully applied to ETo estimation using different subsets of meteorological data, with studies reporting improved accuracy even with fewer input variables^22,23^. For instance, some models using only temperature or solar radiation have achieved competitive performance compared to traditional methods^24,25^. However, most previous studies have focused on either developing a single ML model or optimizing a specific equation, with limited research systematically comparing multiple algorithms under various input feature scenarios.

This study addresses this gap by evaluating the performance of three machine learning algorithms: K-Nearest Neighbors (KNN), Decision Tree (DT), and Random Forest (RF) to estimate ETo under different combinations of climatic inputs. The goal is to identify a balance between estimation accuracy and model simplicity, providing a practical, cost-effective framework for irrigation scheduling and water resource management, particularly in water-scarce agricultural regions.

## Materials and methods

### Study area

**Fig. 1:**
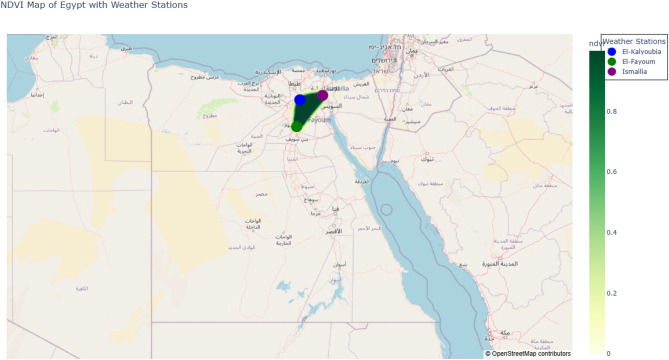
Map of weather stations.

This study was conducted in three agriculturally significant governorates of Egypt—El-Kalyoubia, El-Fayoum, and Ismailia—to capture a broad range of agroecological and climatic conditions (Fig. 1). These sites were deliberately chosen to ensure that the study outcomes are broadly representative of Egypt’s major agricultural zones:

- El-Kalyoubia: Located in the Nile Delta, this region is characterized by fertile alluvial soils, intensive fruit and vegetable production, and a semi-arid climate with relatively high humidity influenced by its proximity to the Nile River.
- El-Fayoum: Situated in a natural depression southwest of Cairo, El-Fayoum represents a transition zone between irrigated agriculture and desert plateaus. The area is irrigated by Nile-fed canals and experiences significant spatial climatic variability.
- Ismailia: Positioned in northeastern Egypt near the Suez Canal, Ismailia has sandy, low-fertility soils and relies heavily on irrigation. It is known for high solar radiation and strong winds, making it suitable for testing water-use estimation models under challenging environmental conditions.

The selected governorates encompass diverse topography, soil types, and cropping systems, providing a robust basis for evaluating reference evapotranspiration (ETo) prediction models in a range of agroecosystems.

### Dataset and preprocessing

Daily meteorological data were collected from three private automated weather stations (Ambient Weather WS-1002-WiFi) installed at the study sites and summarized in Table 1. Each station was equipped with sensors to measure air temperature, relative humidity, wind speed, wind direction, rainfall, and solar radiation. Data were continuously recorded over a three-year period (January 2021–December 2023), producing a total of 3,286 daily observations per station.

**Table 1:** Summarizes the meteorological parameters, units, sensor types, and their relevance to evapotranspiration modeling.

Parameter	Symbol	Unit	Source/Sensor Type	Description and Relevance
Maximum temperature	Tmax	°C	Thermistor sensor, WS-1002-WiFi	Daily max air temperature at 2 m height
Minimum temperature	Tmin	°C	Thermistor sensor, WS-1002-WiFi	Daily min air temperature at 2 m height
Relative humidity (max)	RHx	%	Capacitive humidity sensor	Maximum daily relative humidity
Relative humidity (min)	RHn	%	Capacitive humidity sensor	Minimum daily relative humidity
Wind speed	U2	m s⁻¹	Ultrasonic anemometer, WS-1002-WiFi	Average daily wind speed at 2 m
Wind direction	WD	Degrees	Ultrasonic anemometer	Average daily wind direction
Solar radiation	Rs	MJ m⁻² day⁻¹	Pyranometer sensor	Total daily solar energy received
Rainfall	P	mm	Tipping-bucket rain gauge	Daily total precipitation

**Fig. 2:**
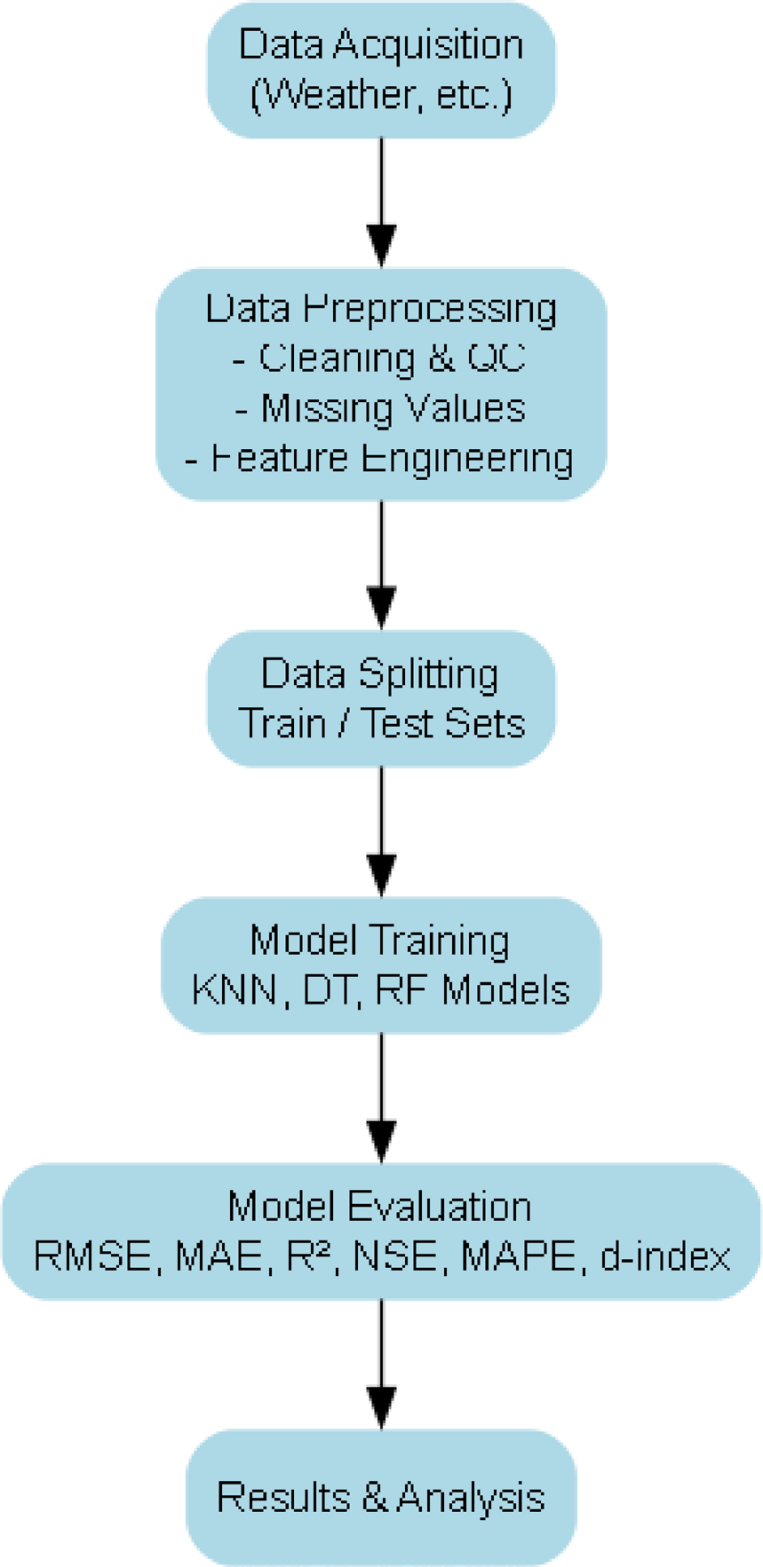
flowchart detailing the methodology.

All meteorological data were initially stored in Microsoft Excel format and processed using Python (v3.10). The preprocessing workflow consisted of three main steps as shown in Fig. 2:

1. Data Cleaning and Imputation: Missing values, which accounted for < 5% of all records, were replaced using mean imputation via the SimpleImputer class from scikit-learn. The same method was applied to both predictor and target variables.
2. Dataset Partitioning: The dataset was divided into training (80%) and testing (20%) subsets using train_test_split with random_state = 42 for reproducibility. A 5-fold cross-validation scheme was implemented during model training and hyperparameter tuning to minimize overfitting.
3. Feature Standardization: All predictor variables were standardized to zero mean and unit variance using the StandardScaler class. Parameters were computed from the training set only, then applied to the test set to prevent data leakage:

1 $$\:z=\frac{x\:-\:\mu\:}{\sigma\:}$$

where x is the original feature, µ is its mean, and σ is its standard deviation (training set only).

#### Autocorrelation analysis

 To assess temporal dependencies in meteorological variables, autocorrelation (ACF) and partial autocorrelation (PACF) functions were computed for each input parameter at lags of 0-365 and 1–30 days, respectively. Results showed strong lag-1 autocorrelation in temperature and relative humidity series, while wind speed and solar radiation displayed weaker short-term dependencies. These findings highlight the temporal persistence of climate variables, which may affect predictive performance and are discussed in Sect. 3.

2 $$\:ACF\left(k\right)\:=\:\frac{\sum\:_{t=k+1}^{N}({x}_{t}\:-\:\overline{x})({x}_{t-k}\:-\:\overline{x})}{\sum\:_{t\:=\:1}^{N}{({x}_{t}\:-\:\overline{x})}^{2}}$$

Where:

- k: Lag order.
- X_t_​: Value of the variable at time t.
- $\:\overline{x}$: Mean of the series.
- N: Number of observations.

Measures the direct correlation between x_t_​ and x_t−k_ after removing the effect of intermediate lags. Estimated using Yule–Walker or regression-based methods:

3 $$\:PACF\left(k\right)\:=\:{{\varnothing}}_{kk}$$

Where:

$\:{\varnothing\:}_{kk}$ ​: The partial regression coefficient of x_t−k_​ when regressing x_t_​ on its lagged values up to k.

#### Multicollinearity assessment

Multicollinearity among input features was evaluated using Pearson correlation coefficients and Variance Inflation Factor (VIF) scores. Tmax and Tmin exhibited strong correlation (*r* ≈ 0.89), while RH variables correlated moderately with temperature (*r* ≈ − 0.65). All VIF scores were below 5, indicating acceptable levels of multicollinearity. Tree-based models (DT, RF) are inherently robust to correlated features, while KNN was standardized to mitigate scale effects. No variables were removed due to multicollinearity to preserve physical interpretability of the Penman–Monteith framework. Quantifies multicollinearity among predictor variables:

4 $$\:{VIF}_{i}=\frac{1}{1\:-\:{R}_{i}^{2}}$$

Where:

- ​$\:{R}_{i}^{2}$: Coefficient of determination obtained by regressing predictor x_i_ on all other predictors.
- Interpretation:
  - VIF > 10: Strong multicollinearity.
  - VIF < 5: Acceptable range.

### Reference evapotranspiration calculation

Reference evapotranspiration (ETo) was calculated using the FAO Penman–Monteith equation^26^:

5 $$\:{ET}_{o}=\:\frac{0.408\:\varDelta\:\:\left({R}_{n}-G\right)+\gamma\:\frac{900}{T+273}{U}_{2}({e}_{s}-{e}_{a})}{\varDelta\:+\:\gamma\:\:(1+0.34\:{U}_{2})}$$

Where:

ET_o_ reference evapotranspiration [mm day^−1^],

R_n_ net radiation at the crop surface [MJ m^−2^ day^−1^],

G soil heat flux density [MJ m^−2^ day^−1^,

T mean daily air temperature at 2 m height [°C],

u_2_ wind speed at 2 m height [m s^−1^],

e_s_ saturation vapour pressure [kPa],

e_a_ actual vapour pressure [kPa],

e_s_ - e_a_ saturation vapour pressure deficit [kPa],

Δ slope vapour pressure curve [kPa °C^−1^],

γ psychrometric constant [kPa °C^−1^].

### Map of the study area

Figure 1 shows a publication-quality map of the study area, including station locations, topographic features (digital elevation model), vegetation cover (NDVI-based land classification), and administrative boundaries. This representation provides essential spatial context for understanding agroclimatic variability across the selected regions.

### Machine learning models

#### K-nearest neighbors (KNN)

The KNN algorithm predicts target values based on the responses of its k nearest neighbors in feature space. The Manhattan distance metric was applied:

6 $$\:d\left(x,y\right)={\sum\:}_{i=1}^{n}\lceil{x}_{i}-{y}_{i}\rceil$$

where *d*(x, y) is the distance between vectors x and y in n-dimensional space. Hyperparameters: k ∈ [1,10], optimized via 5-fold cross-validation based on minimum RMSE.

#### Decision tree (DT)

The DT algorithm partitions the feature space into homogeneous regions via hierarchical, rule-based splits. Maximum tree depth (*d*_*max*_​) was tuned between 1 and 10 to prevent overfitting.

#### Random forest (RF)

RF is an ensemble method combining multiple decision trees, reducing overfitting through bootstrapping and random feature selection. Hyperparameters included:

- Number of trees (n_estimatorsn_ ​): [10, 50, 100, 200, 300, 400, 500, 600, 700, 800]
- Maximum depth (d_max_​): [1–10]

### Model scenarios

Four modeling scenarios were implemented to assess the contribution of different meteorological inputs:

1. Full feature set: *T*, *R*_*s*_​, *U*_*2*_​, *RH*.
2. Three feature combinations: e.g., *T*, *RH*, *U*_*2*_; *T*, *RH*, *R*_*s*_​.
3. Two feature combinations: e.g., *T*, *R*_*s*_; *RH*, U_2​_.
4. Single-feature models: *T*, *R*_*s*_, *U*_*2*_​, or *RH* individually.

This structure reveals the most influential predictors for ETo estimation and evaluates model robustness under data-limited conditions.

### Model evaluation metrics

The predictive performance of the machine learning models was rigorously assessed using a comprehensive set of statistical indices. These metrics evaluate both the accuracy and reliability of predictions relative to observed reference evapotranspiration (ETo) values.

#### Error-based metrics

The Root Mean Square Error (RMSE) and Mean Absolute Error (MAE) quantify the magnitude of prediction errors, while the coefficient of determination (R^2^) measures the proportion of variance in the observed data explained by the model^27,28^:

7 $$\:RMSE=\:\sqrt{\frac{1}{n}\sum\:_{i=1}^{n}{\left({O}_{i}-{P}_{i}\right)}^{2}}$$

8 $$\:MAE=\frac{1}{n}\sum\:_{i=1}^{n}\left(\left|{O}_{i}-{P}_{i}\right|\right)$$

9 $$\:{R}^{2}\:=\:1\:-\:\frac{{\sum\:}_{i=1}^{n}{({O}_{i}-{P}_{i})}^{2}}{{\sum\:}_{i=1}^{n}{({O}_{i}-\overline{O})}^{2}}$$

where P_i_​ and O_i_​ are the predicted and observed values, $\:\overline{O}$ is the mean of observed values, and n is the sample size.

#### Bias and relative error metrics

To identify systematic bias and express error magnitude as a percentage of observed values, the Mean Bias Error (MBE) was computed:

10 $$\:MBE=\:\frac{1}{n}\sum\:_{i=1}^{n}\left({P}_{i}-{O}_{i}\right)$$

#### Model efficiency and agreement indices

The Nash–Sutcliffe Efficiency (NSE) and Kling-Gupta Efficiency (KGE) provide robust measures of predictive skill and agreement between predicted and observed values:

11 $$\:NSE\:=\:1-\frac{\sum\:_{i=1}^{n}{({P}_{i}-{O}_{i})}^{2}}{\sum\:_{i=1}^{n}{({O}_{i}-\overline{O})}^{2}}\:\:\:\:\:\:\:$$

The Kling-Gupta Efficiency (KGE) was chosen as the primary metric for model evaluation due to its ability to integrate correlation, bias, and variability errors into a single comprehensive measure. The KGE is defined as:

12 $$\:KGE\:=\:1\:-\:\sqrt{{(r-1)}^{2}+{(\beta\:-1)}^{2}+{(\gamma\:-1)}^{2}}$$

where:

- r is the Pearson correlation coefficient between observed and simulated values, representing linear correlation,
- $\:\beta\:=\frac{\overline{S}}{\overline{O}}\:$is the bias ratio between the mean simulated values ($\:\overline{S}$) and mean observed values ($\:\overline{O}$),
- $\:\gamma\:=\frac{{Cv}_{s}}{{CV\:}_{O}}$ is the variability ratio between the coefficient of variation (CV) of simulated values (CV_s_ ​) and observed values (CV​_o_).

Each model was trained and tested using a cross-validation strategy to ensure robustness and generalizability of the results. The mean KGE values along with their standard deviations (represented as error bars) were computed for each scenario. To statistically assess differences between model-scenario performances, group letters (A–O) were assigned based on post-hoc multiple comparison tests (e.g., Tukey’s HSD), with shared letters indicating no statistically significant difference at the 95% confidence level.

A model achieves perfect predictive performance when RMSE, MAE, and MBE approach zero, while R^2^, NSE, and KGE approach unity.

This expanded evaluation framework provides a holistic assessment of model performance, capturing absolute error, bias, percentage-based error, and overall predictive agreement across different input feature scenarios.

## Results and discussions

### Temporal dependency analysis through ACF and PACF

To examine the temporal characteristics of the meteorological variables used in the modeling process, autocorrelation (ACF) and partial autocorrelation (PACF) analyses were performed. These analyses were applied to maximum temperature (Tx), minimum temperature (Tn), wind speed at 2 m (U2), solar radiation (Rs), maximum relative humidity (HRx), minimum relative humidity (HRn), and reference evapotranspiration (ETrs).

**Fig. 3:**
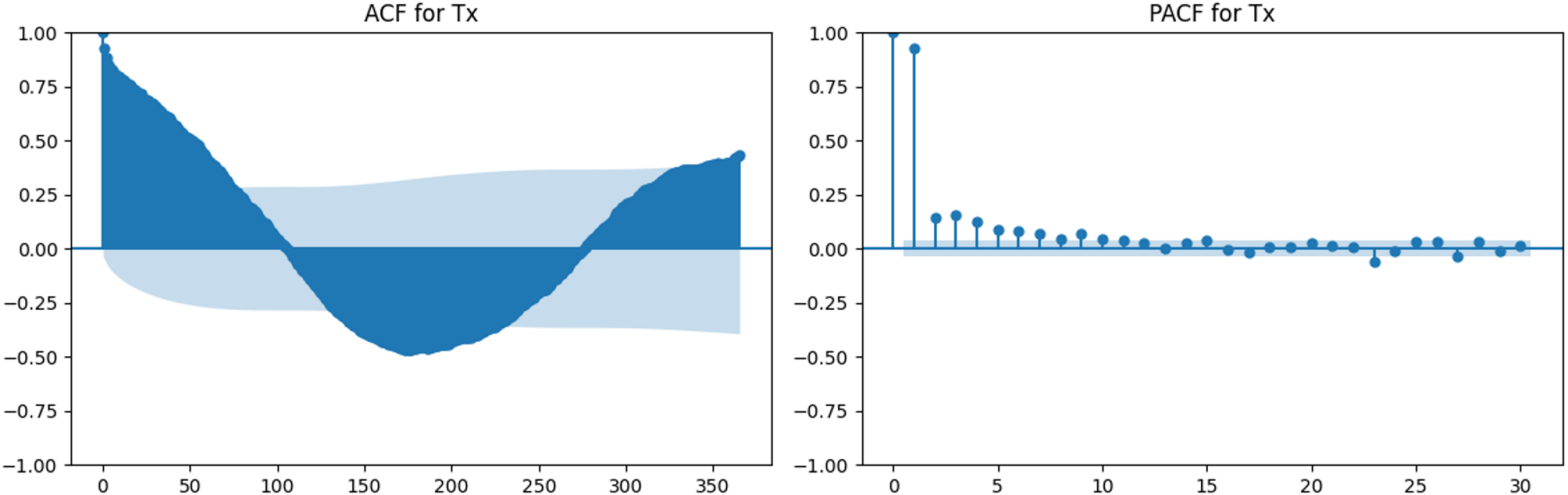
ACF and PACF plots for maximum temperature (Tx).

**Fig. 4:**
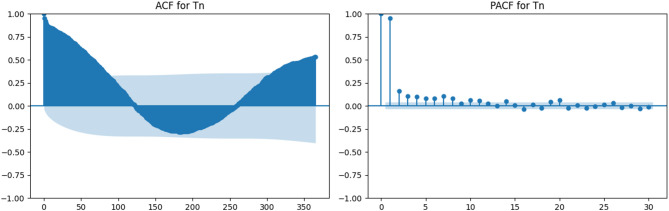
ACF and PACF plots for minimum temperature (Tn).

**Fig. 5:**
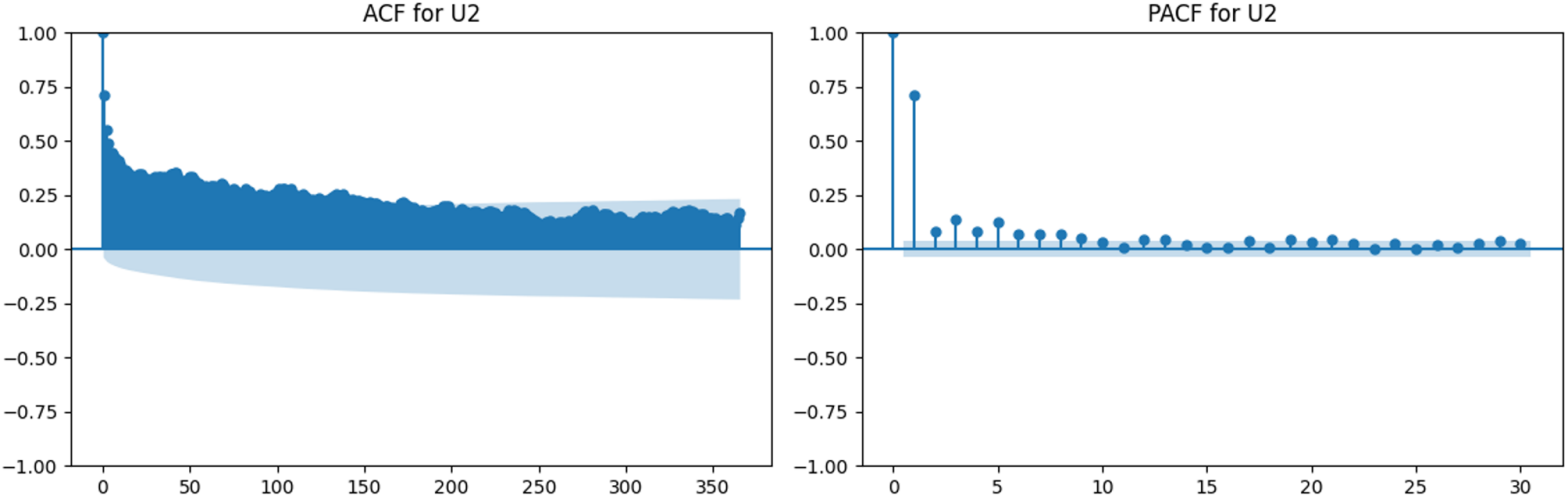
ACF and PACF plots for wind speed at 2 m (U2).

**Fig. 6:**
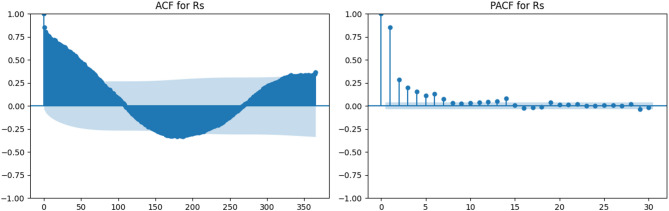
ACF and PACF plots for solar radiation (Rs).

**Fig. 7:**
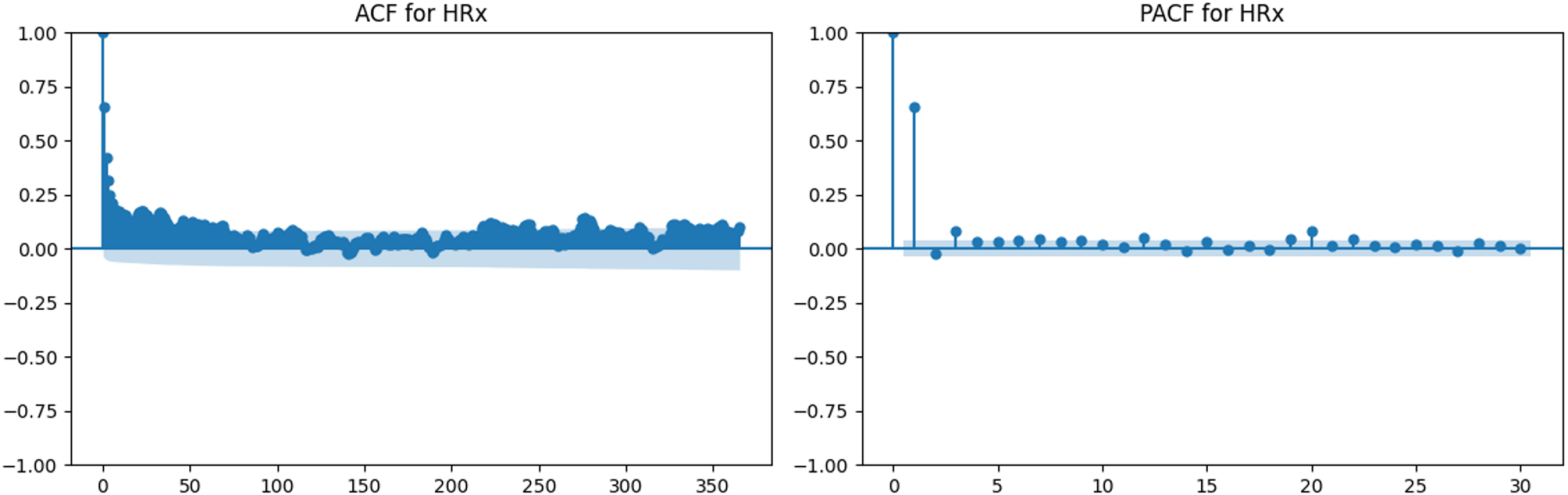
ACF and PACF plots for maximum relative humidity (HRx).

**Fig. 8:**
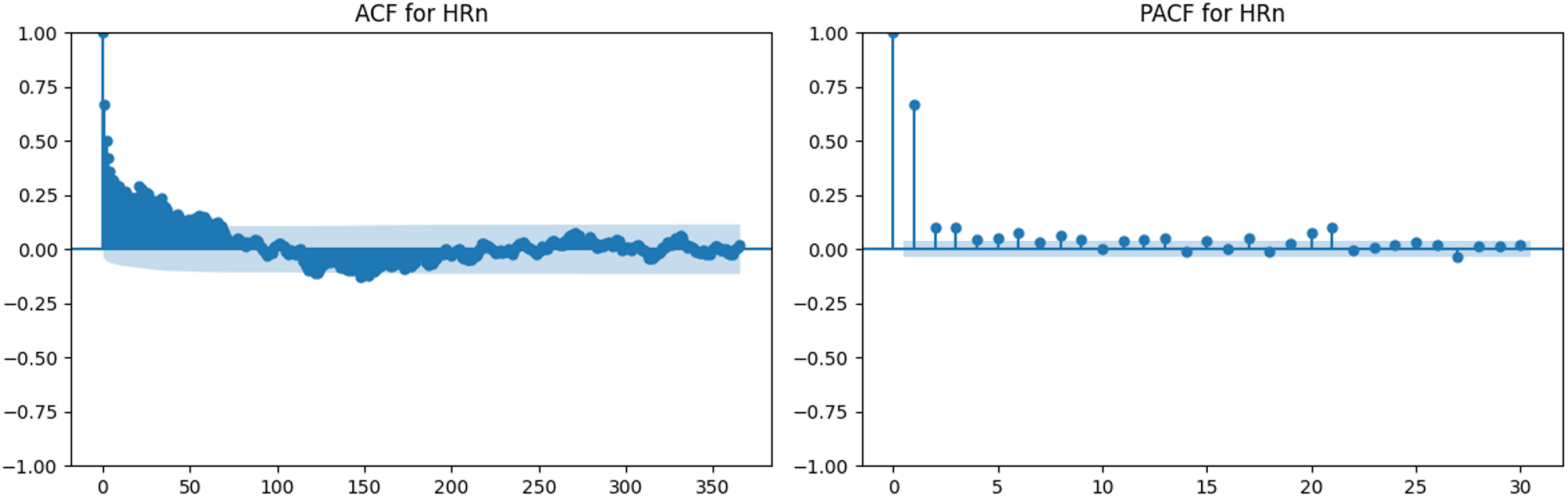
ACF and PACF plots for minimum relative humidity (HRn).

**Fig. 9:**
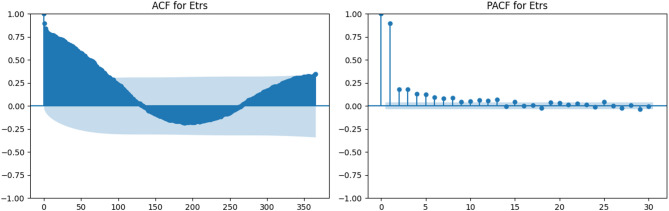
ACF and PACF plots for reference evapotranspiration (ETrs).

Figures 3, 4, 5, 6, 7, 8 and 9 display the ACF and PACF plots for each variable. As shown in Figs. 3 and 4, both Tx and Tn exhibit strong seasonal behavior, with ACF patterns displaying a sinusoidal structure and slowly decaying correlations. Their PACF plots reveal significant correlations at lag 1 and lag 2, followed by a rapid decline, indicating that while these variables exhibit long-term seasonal dependencies, short-term autoregressive influence is relatively limited.

In contrast, U2 (Fig. 5) shows a sharp initial drop in the ACF and weak persistence beyond lag 10, while its PACF indicates significance only at the first few lags. This suggests limited temporal memory and minimal autocorrelation in the wind speed data.

Solar radiation (Fig. 6) also displays a pronounced seasonal pattern similar to temperature, with high autocorrelation at lags associated with the annual cycle. The PACF for Rs confirms significant short-term lags (1–3), supporting the relevance of recent values in predictive modeling.

Relative humidity (Figs. 7 and 8) demonstrates substantially weaker autocorrelation. Both HRx and HRn exhibit a rapid decline in the ACF and limited significant lags in the PACF, indicating near-random temporal behavior. This justifies treating these variables as independent across daily time steps in non-sequential machine learning models.

Reference evapotranspiration (ETrs) follows a seasonal autocorrelation pattern (Fig. 9), with high ACF values persisting over annual cycles, and significant PACF lags at positions 1–5. This suggests that ETrs, while seasonally dependent, can be effectively modeled using daily meteorological inputs without requiring explicit time-series modeling frameworks.

These findings collectively support the application of machine learning models in a static (non-temporal) framework, as most variables exhibit weak short-term temporal dependencies that can be implicitly captured by data-driven models.

### Correlation analysis of predictor variables

**Fig. 10:**
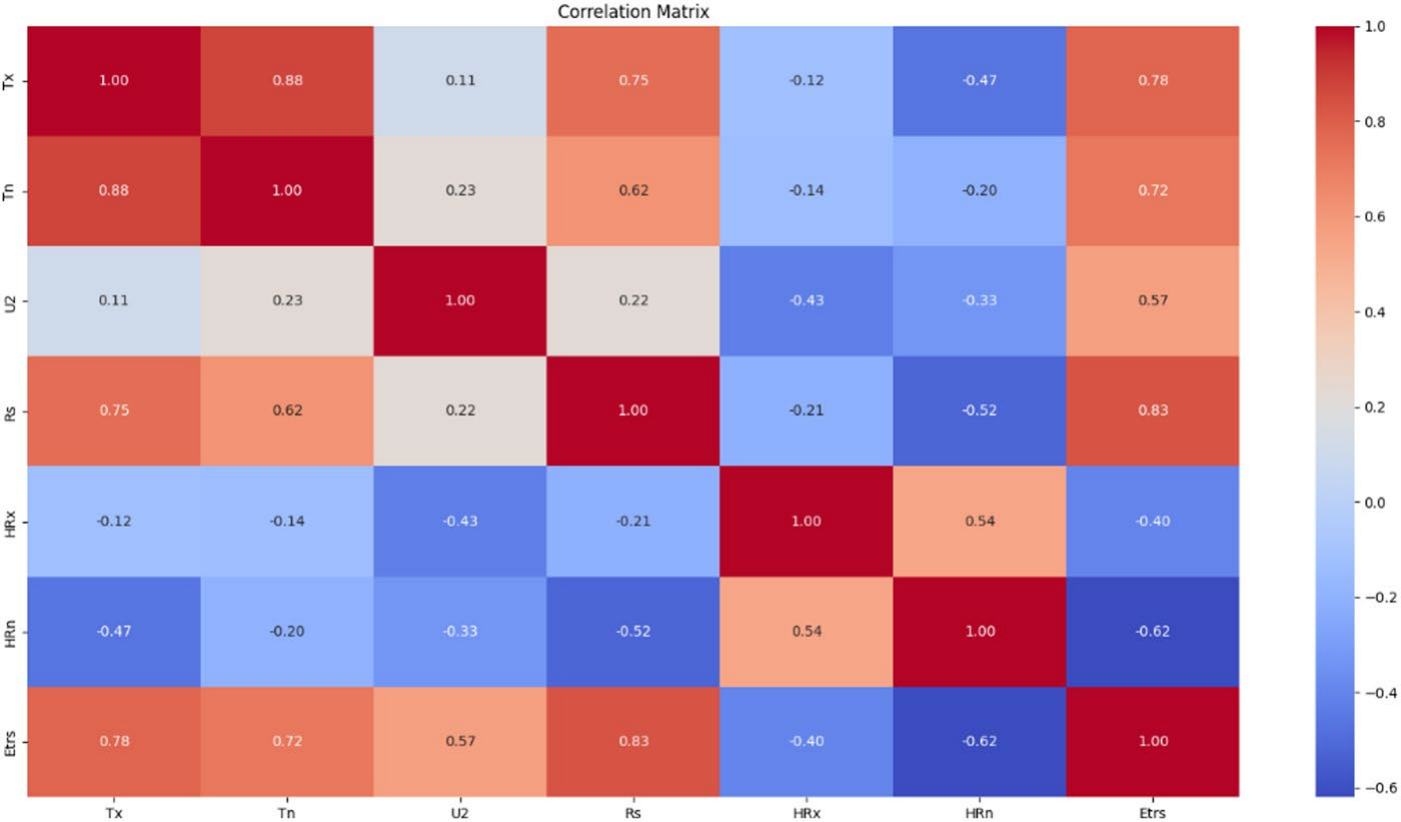
Correlation matrix among meteorological variables and ETrs.

The correlation matrix presented in Fig. 10 provides insight into the linear relationships among the input variables and ETrs. Solar radiation (Rs) exhibited the highest positive correlation with ETrs (*r* = 0.83), followed by maximum temperature (*r* = 0.78) and minimum temperature (*r* = 0.72). These strong correlations highlight the critical role of radiative and thermal energy in driving evapotranspiration.

Wind speed (U2) showed a moderate positive correlation with ETrs (*r* = 0.57), reflecting its contribution to vapor transport and surface moisture removal. In contrast, maximum and minimum relative humidity (HRx and HRn) demonstrated negative correlations with ETrs (*r* = − 0.40 and *r* = − 0.62, respectively), indicating that higher humidity suppresses evapotranspiration by reducing the vapor pressure deficit.

Strong inter-variable correlations were also observed, particularly between Tx and Tn (*r* = 0.88), and between HRx and HRn (*r* = 0.54), suggesting potential multicollinearity. These relationships were considered during feature selection and scenario design to ensure model generalizability and reduce redundancy.

Overall, the correlation analysis guided the selection of the most informative features for predictive modeling. Rs, Tx, and Tn emerged as the most influential variables, while U2 and humidity metrics provided complementary information useful in capturing complex evapotranspiration dynamics.

### Model evaluation across scenarios

**Fig. 11:**
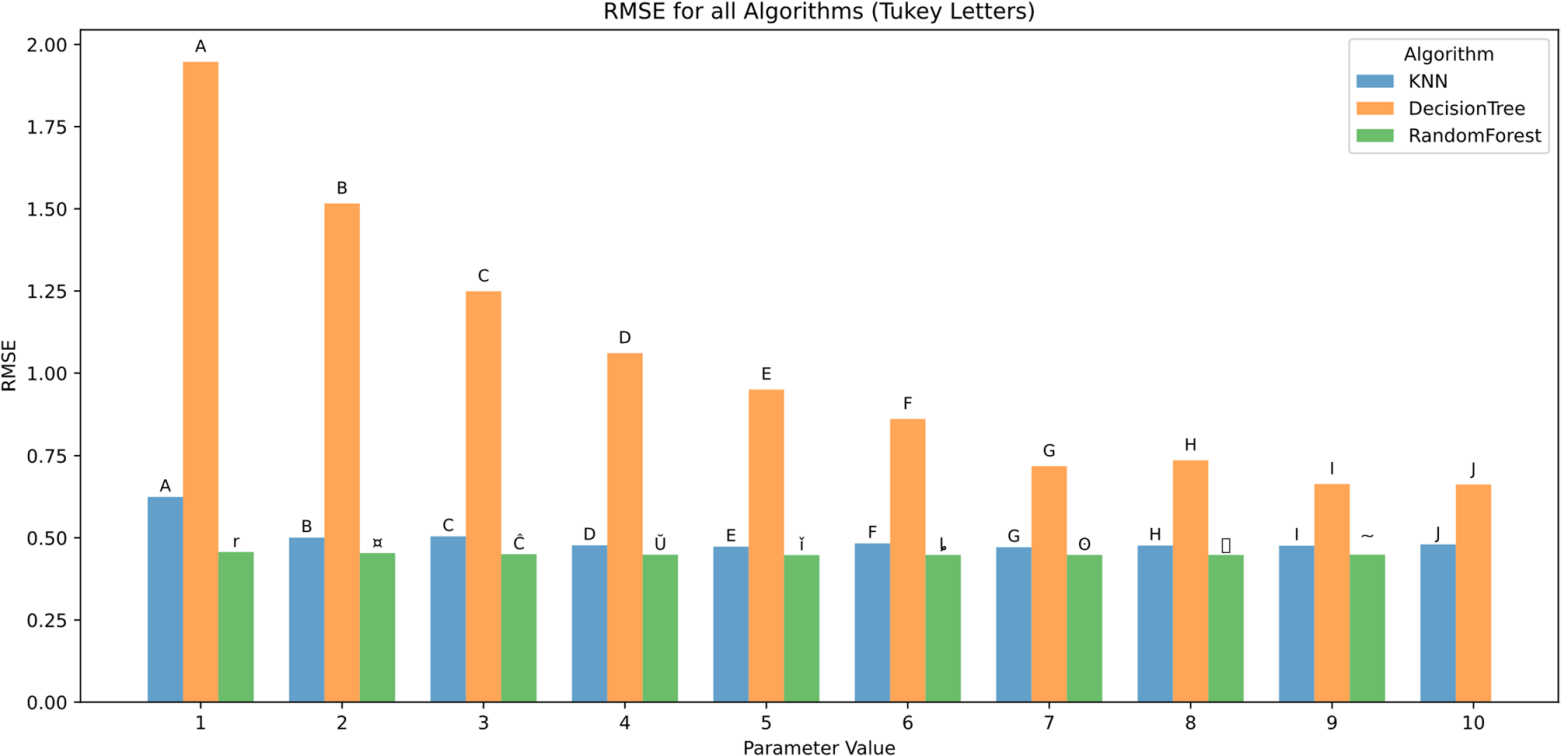
RMSE across hyperparameters and feature scenarios.

**Fig. 12:**
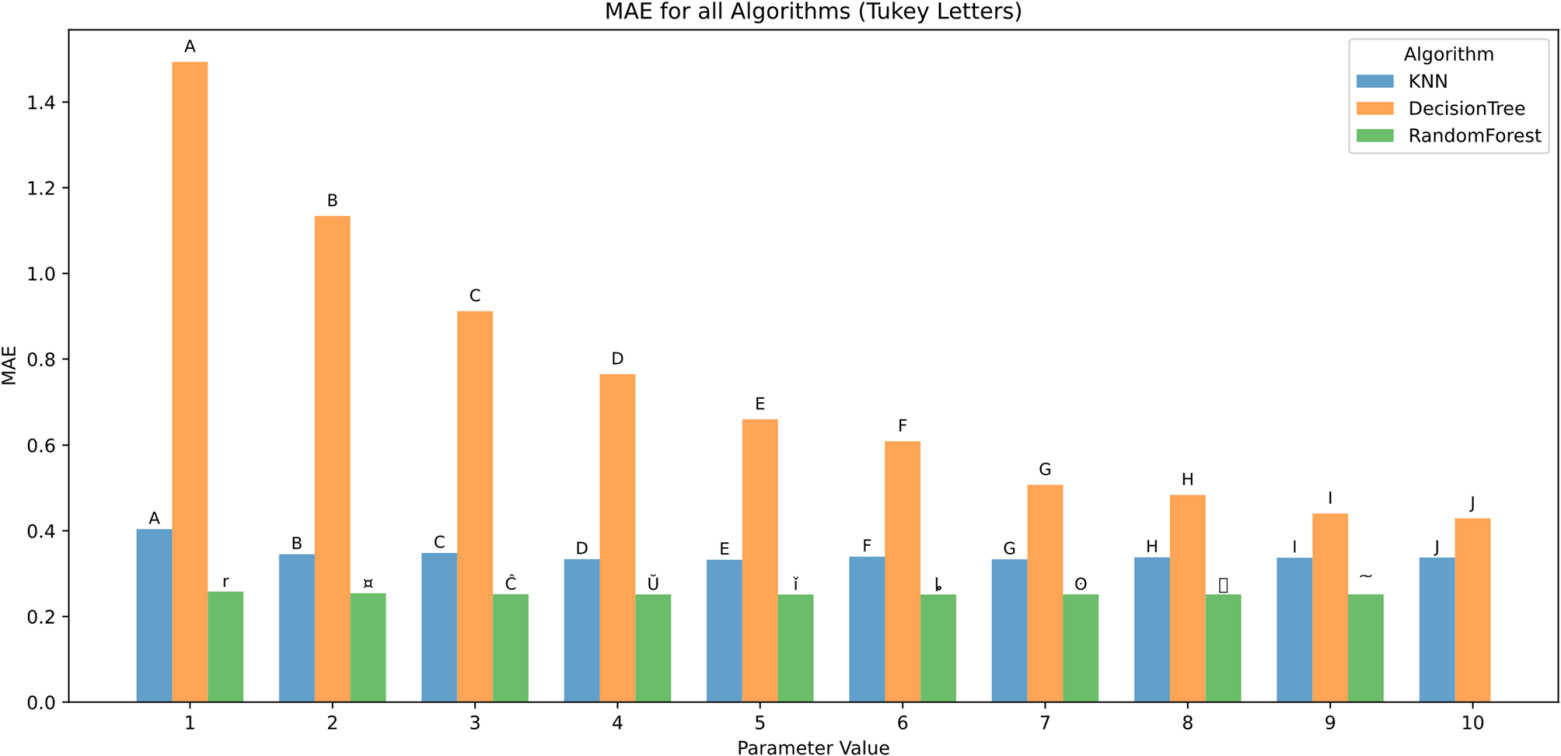
MAE across hyperparameters and feature scenarios.

**Fig. 13:**
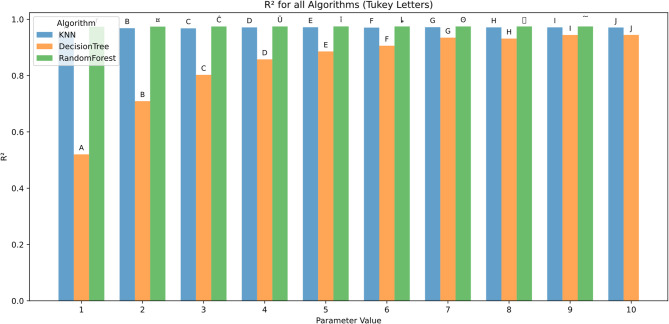
R² across hyperparameters and feature scenarios.

**Fig. 14:**
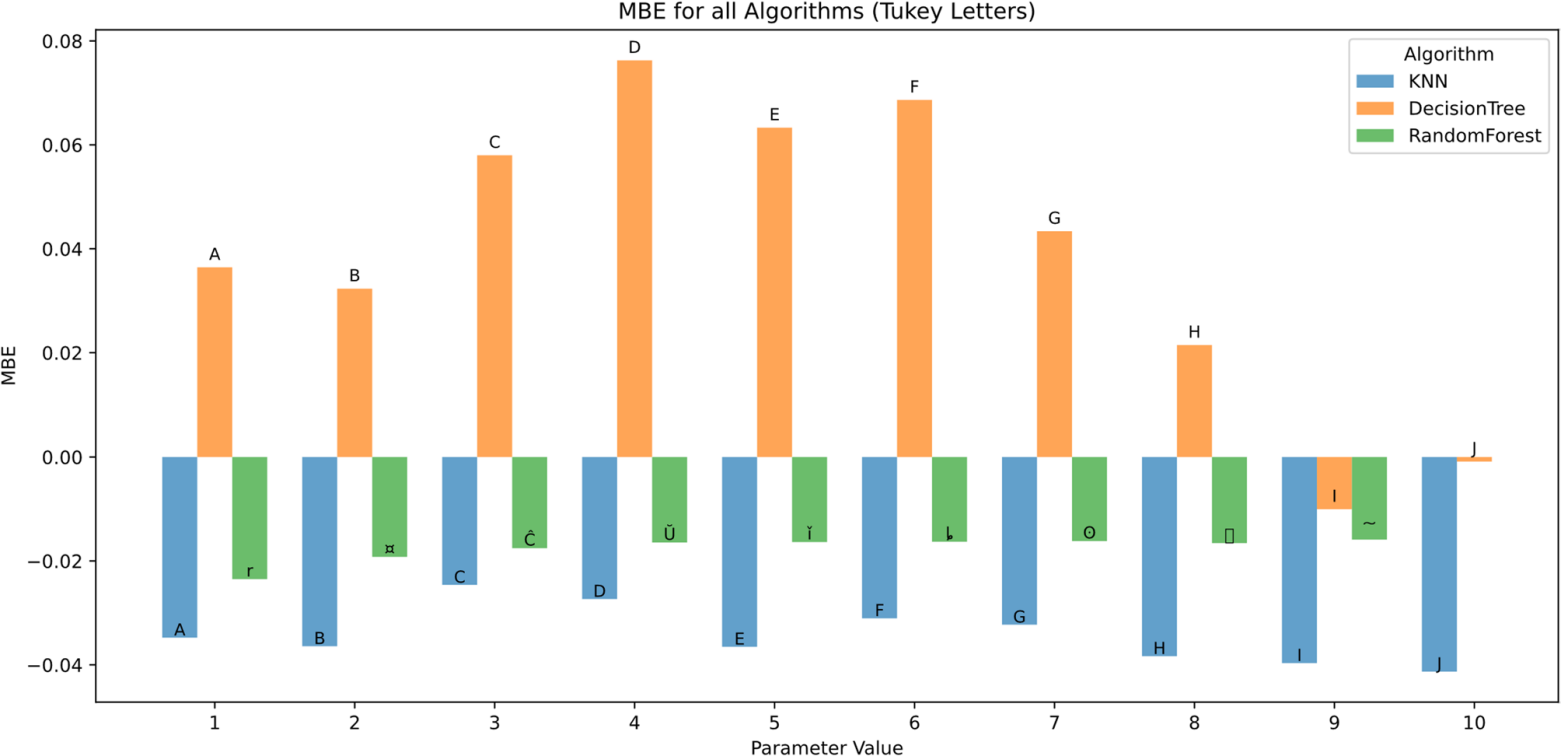
MBE across hyperparameters and feature scenarios.

**Fig. 15:**
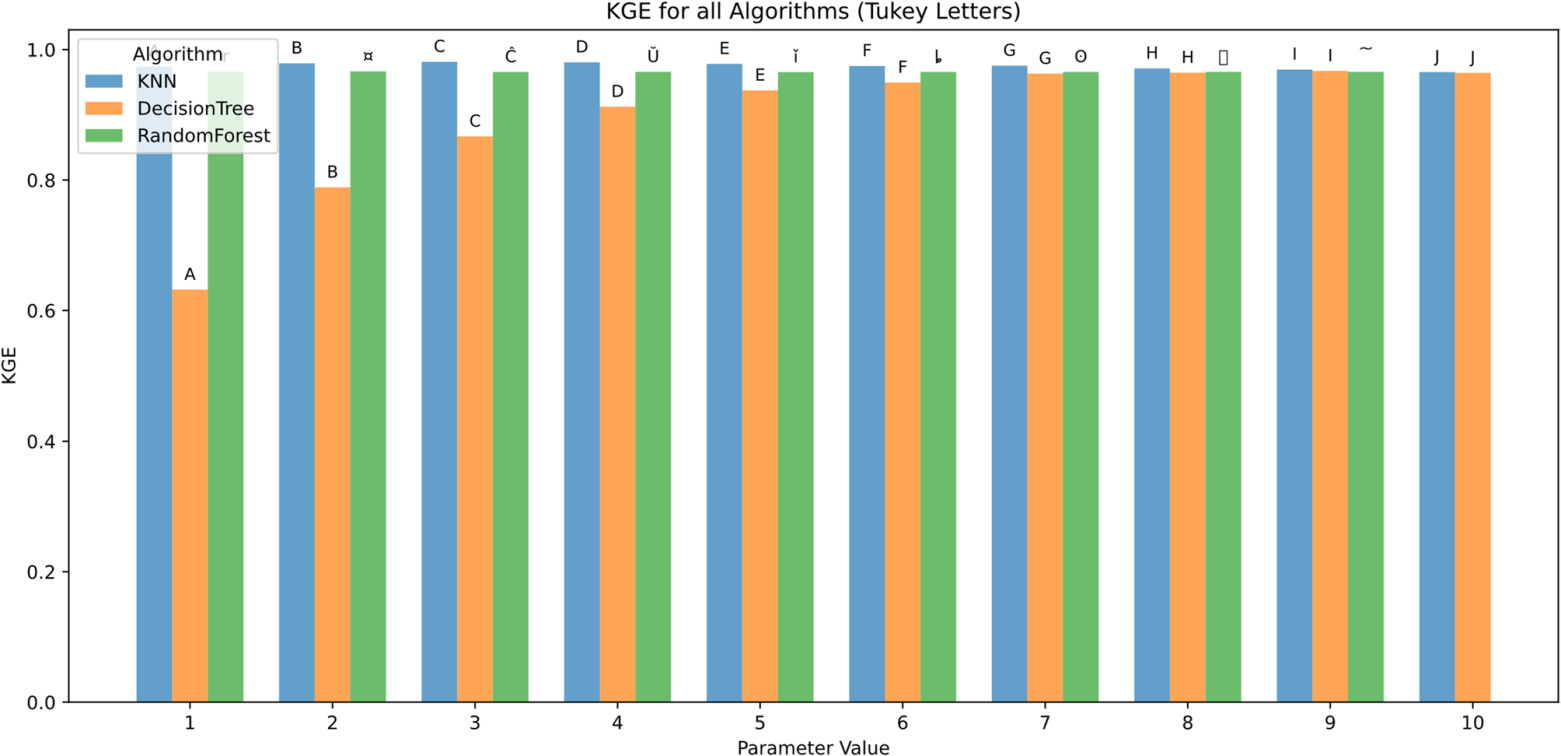
KGE across hyperparameters and feature scenarios.

**Fig. 16:**
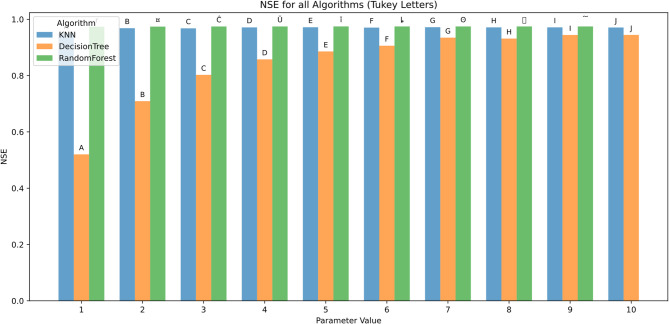
NSE across hyperparameters and feature scenarios.

**Fig. 17:**
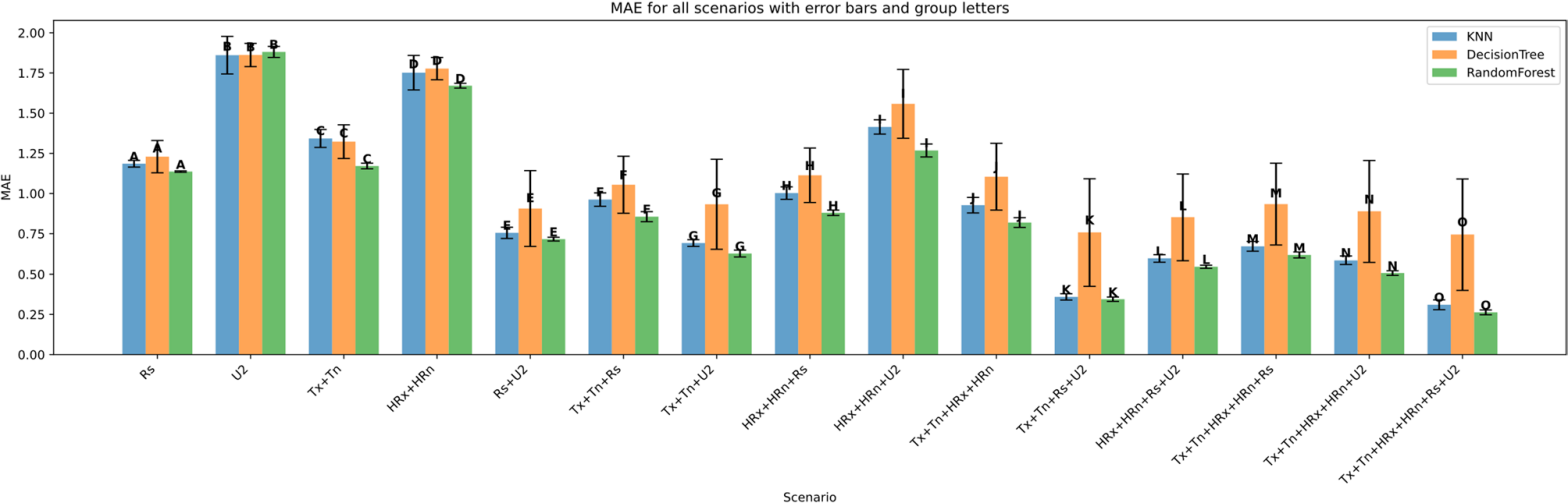
RMSE across predictor scenarios.

The performance of three machine learning models—K-Nearest Neighbors (KNN), Decision Tree (DT), and Random Forest (RF)—was evaluated under four input feature scenarios: (i) all features combined, (ii) three-feature combinations, (iii) two-feature combinations, and (iv) single-feature inputs. Model performance was assessed using Root Mean Squared Error (RMSE), Mean Absolute Error (MAE), the coefficient of determination (R²), Mean Bias Error (MBE), Nash-Sutcliffe Efficiency (NSE), and KGE. Summary results are presented in Figs. 11, 12, 13, 14, 15, 16 and 17.

#### Performance across parameter values

The effect of algorithmic parameter tuning was assessed for KNN, DT, and RF using ten discrete parameter levels (1–10). Evaluation metrics included RMSE, MAE, R², MBE, KGE, and NSE. Tukey’s HSD test (α = 0.05) was applied for statistical grouping.

RMSE As illustrated in Fig. 11, all algorithms exhibited decreasing RMSE with increasing parameter values. Decision Tree consistently produced the highest RMSE values, starting at 1.97 (group A) for parameter 1 and improving to 0.65 (group J) at parameter 10. KNN demonstrated a gradual reduction in RMSE, ranging from 0.64 (A) to 0.45 (J). Random Forest yielded the lowest RMSE values across all parameter settings, ranging from 0.47 (group r) to 0.43 (group J).

Significant differences were observed between algorithms at each parameter value. Random Forest outperformed Decision Tree at all levels and achieved statistical groupings that indicate significantly better performance (groups r–J vs. A–J). Performance gains plateaued after parameter value 7 for all models.

Figure 12 shows MAE trends mirrored those of RMSE. Decision Tree exhibited the highest MAE at parameter 1 (1.48, group A) and the lowest at parameter 10 (0.43, group J). KNN showed improved performance from 0.41 (group A) to 0.32 (group J), while Random Forest again yielded the lowest MAE, decreasing from 0.24 (group r) to 0.22 (group J).

The Random Forest consistently fell into the lowest statistical groupings, indicating its superior accuracy in minimizing absolute prediction error.

Figure 13 shows coefficient of determination (R²) values improved with increasing parameter settings. Random Forest achieved near-perfect R² values across all levels (0.96 to 0.99, groups ¤–J). KNN followed closely, reaching R² = 0.99 (group J) at parameter 10. In contrast, Decision Tree exhibited notably lower R² values, especially at lower parameter levels (0.52 at parameter 1, group A), with maximum improvement to 0.94 (group J) at parameter 10.

These results indicate better model fit and generalization by Random Forest and KNN, especially at higher parameter levels.

.

Mean Bias Error (MBE) analysis (Fig. 14) revealed systematic prediction biases. KNN and Random Forest predominantly exhibited negative MBE values, suggesting a tendency to underpredict, with MBE values ranging from − 0.04 to − 0.01. Conversely, Decision Tree showed a positive bias, with peak MBE at 0.077 (group D) at parameter 4.

This indicates a structural tendency of Decision Trees toward overestimation, particularly at mid-range parameter values.

Figure 15 shows model performance, as measured by the Kling-Gupta Efficiency (KGE), improved consistently with increasing parameter values. At the lowest parameter setting (1), KNN and Random Forest performed moderately (KGE ≈ 0.85), while Decision Tree lagged significantly (KGE ≈ 0.65), marked by group A. As the parameter value increased, all models showed steady improvement. By parameter value 6, KGE values exceeded 0.98 across models, with Random Forest slightly outperforming others, and statistical differences becoming negligible (group F and beyond). From parameter value 7 onward, all three models achieved near-perfect performance (KGE > 0.99) and shared the same statistical groupings (G–J), indicating no significant differences. Overall, Random Forest maintained the most consistent top-tier performance, while Decision Tree showed the greatest relative improvement as parameter values increased.

Figure 16 shows model performance, measured by the Nash–Sutcliffe Efficiency (NSE), followed a trend similar to that observed for KGE. At parameter value 1, performance was lowest, particularly for the Decision Tree model, which achieved an NSE of approximately 0.55 (group A), while KNN and Random Forest scored higher (around 0.85). As the parameter value increased, NSE improved for all models. From parameter value 6 onward, NSE values exceeded 0.95 for all models, with Random Forest showing slightly higher stability. By parameter value 9 and 10, all models achieved NSE values above 0.98, and statistical groupings converged to group J, indicating no significant performance differences. Overall, Random Forest again showed the most consistent and high performance across all parameter settings, while Decision Tree showed the largest improvement range.

#### Performance across feature scenarios

The performance of the three machine learning models—K-Nearest Neighbors (KNN), Decision Tree (DT), and Random Forest (RF)—was evaluated in terms of RMSE, MAE, R², MBE, KGE, and NSE﻿ across 13 input feature scenarios. Error bars indicate the variability of all terms, and group letters denote statistically significant differences (*p* < 0.05) among the scenarios for each model.

The performance of the three models—K-Nearest Neighbors (KNN), Decision Tree, and Random Forest—was evaluated across multiple scenarios using Root Mean Square Error (RMSE) as the accuracy metric. Overall, Random Forest consistently outperformed the other models, especially in scenarios combining multiple features. For instance, in the most comprehensive scenario involving all variables (Tx + Tn + HRx + HRn + Rs + U2), both Random Forest and KNN achieved the lowest RMSE of approximately 0.5 (group letter O), indicating superior predictive accuracy.

In simpler scenarios such as Rs, KNN slightly outperformed the others with an RMSE around 1.6 (group A). However, as additional features were incorporated, Random Forest showed clear advantages. For example, in scenarios like Tx + Tn + U2 and HRx + HRn + Rs, Random Forest achieved RMSE values as low as 0.9 and 1.2, outperforming KNN and Decision Tree, which recorded higher errors.

Decision Tree generally exhibited the highest RMSE across scenarios, particularly in complex combinations involving Tx and U2, with RMSE often exceeding 1.5. This suggests it is less effective in capturing complex interactions between features compared to the other models.

Error bars representing the standard deviation across multiple runs reveal that Random Forest predictions were not only more accurate but also more stable, showing smaller variance than both KNN and Decision Tree. KNN demonstrated competitive performance in several scenarios but with slightly higher variability, while Decision Tree showed the greatest inconsistency.

Statistical significance indicated by group letters (ranging from A to O) confirms that the differences in RMSE among models are meaningful. Random Forest dominated the higher-ranked groups (M through O) in scenarios with many combined features, while KNN excelled in simpler cases. Decision Tree’s grouping often overlapped with KNN but generally fell behind Random Forest.

These findings highlight Random Forest’s robustness and ability to leverage multiple feature interactions effectively, resulting in both more accurate and consistent predictions compared to KNN and Decision Tree.

**Fig. 18:**
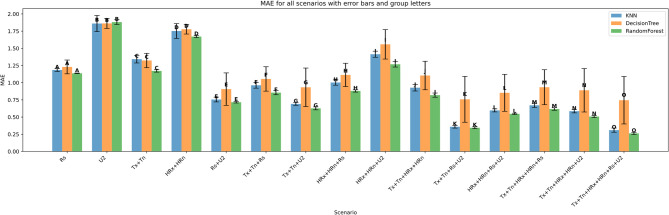
MAE across predictor scenarios.

Figure 18 shows the evaluation of the models using Mean Absolute Error (MAE) across various scenarios reveals trends consistent with the RMSE findings. Random Forest consistently delivers the lowest MAE values in most scenarios, particularly in those involving multiple combined features. For example, in the most comprehensive scenario (Tx + Tn + HRx + HRn + Rs + U2), Random Forest achieves an MAE of approximately 0.3 (group letter O), reflecting highly accurate predictions. KNN shows competitive performance in simpler scenarios such as Rs, with an MAE around 1.2 (group A), but generally lags behind Random Forest as more features are introduced. Decision Tree tends to have the highest MAE values across scenarios, especially when the number of features increases, indicating less precise predictions.

Error bars representing variability further emphasize Random Forest’s superior stability, with smaller error margins compared to the more variable performances of KNN and Decision Tree. Statistical groupings reinforce these observations, with Random Forest dominating the top-performing groups in complex scenarios, while KNN and Decision Tree tend to share overlapping groups with lower performance rankings.

**Fig. 19:**
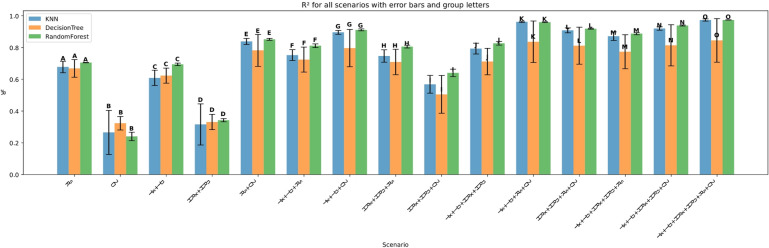
R² across predictor scenarios.

Figure 19 shows the coefficient of determination (R^2^) was used to evaluate the goodness of fit of the models across the various scenarios. Consistent with RMSE and MAE results, Random Forest generally achieved the highest R^2^ values, indicating superior explanatory power and prediction accuracy. For example, in complex scenarios such as Tx + Tn + HRx + HRn + Rs + U2, Random Forest attained an R^2^ close to 0.98 (group letter O), reflecting near-perfect model fit. KNN followed closely, particularly in simpler scenarios like Rs with R^2^ ≈ 0.7 (group A) but showed more variability in more complex combinations. Decision Tree consistently exhibited the lowest R^2^ values and larger error bars, highlighting its limited capacity to capture complex relationships within the data.

Error bars denoting standard deviation across multiple runs again confirm Random Forest’s stability and reliability compared to the more inconsistent performances of KNN and Decision Tree. The group letters corroborate that Random Forest holds significant statistical advantage in the majority of the scenarios, especially as the number of features increases.

**Fig. 20:**
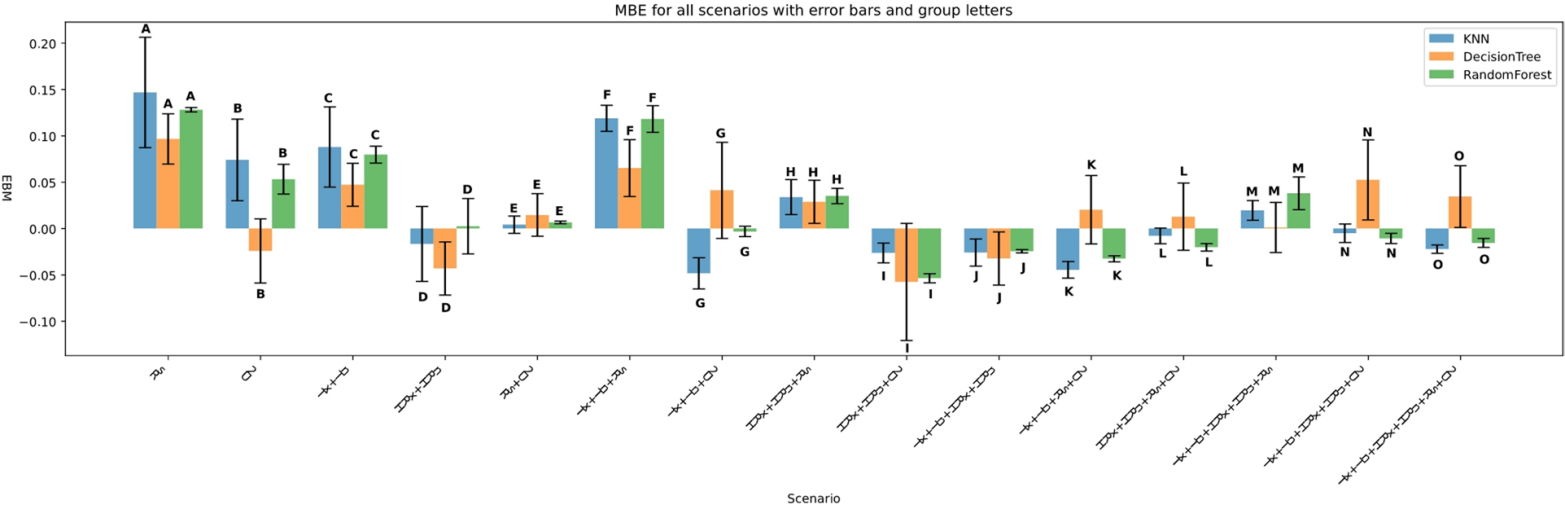
MBE across predictor scenarios.

Figure 20 depicts MBE analysis across different scenarios reveals the bias direction and magnitude in the models’ predictions. Random Forest generally shows minimal bias, with MBE values close to zero in most scenarios, especially in complex feature combinations such as Tx + Tn + HRx + HRn + Rs + U2 (MBE close to 0, group letter O). KNN tends to exhibit a slight positive bias in simpler scenarios like Rs and Tx + Tn, while Decision Tree shows more variable bias, sometimes overestimating and other times underestimating, especially in scenarios involving U2 and Tx + Tn + HRx + HRn.

Error bars indicate that Random Forest maintains a consistent and stable bias across runs, reinforcing its reliability. Conversely, Decision Tree displays larger variability in bias, indicating less dependable predictions.

**Fig. 21:**
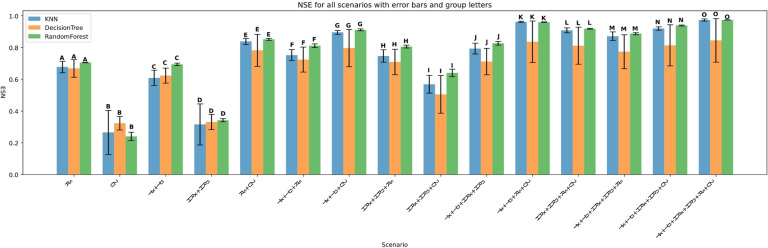
NSE across predictor scenarios.

Figure 21 presents the NSE metric, which measures the predictive skill of the models relative to the observed mean, further supports previous findings. Across all scenarios, Random Forest consistently achieves the highest NSE values, often exceeding 0.9 in complex feature combinations like Tx + Tn + HRx + HRn + Rs + U2 (group O), indicating excellent model performance. KNN closely follows, particularly in simpler scenarios such as Rs with NSE around 0.7 (group A). Decision Tree generally displays the lowest NSE values and higher variability, indicating weaker predictive skill.

Error bars confirm Random Forest’s superior stability, showing lower variance compared to KNN and Decision Tree. The group letters further emphasize the statistical significance of Random Forest’s better performance across most scenarios.

**Fig. 22:**
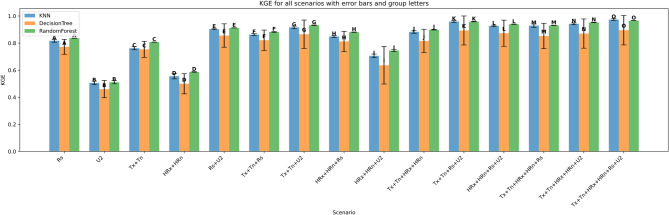
KGE d across predictor scenarios.

The results in Fig. 22 show that model performance improved with the inclusion of more meteorological variables. The highest KGE was achieved by the Random Forest model (0.97) using the full input set (Tx + Tn + HRx + HRn + Rs + U2), followed closely by KNN (0.96) and Decision Tree (0.91). In contrast, the lowest performance was observed in the U2-only scenario, where Random Forest and KNN scored 0.50 and Decision Tree 0.45. Moderate results were seen in configurations like Tx + Tn + Rs (Random Forest: 0.88) and Rs + U2 (0.91). Overall, Random Forest consistently outperformed the other models across all scenarios, with KNN close behind in more complex input combinations. Decision Tree had the weakest performance, particularly in limited-input scenarios.

### Predicted vs. observed reference evapotranspiration (ETo)

The predictive performance of three machine learning models K-Nearest Neighbors (KNN), Decision Tree (DT), and Random Forest (RF) was evaluated for estimating reference evapotranspiration (ETo) under five distinct scenarios (S1 to S5). Key performance metrics including Root Mean Square Error (RMSE), Mean Absolute Error (MAE), Coefficient of Determination (R²), Mean Bias Error (MBE), Nash-Sutcliffe Efficiency (NSE), and KGE were used to assess model accuracy by comparing predicted against observed ETo values.

#### Predicted vs. observed ETo results for scenario 1 (S1)

**Fig. 23:**
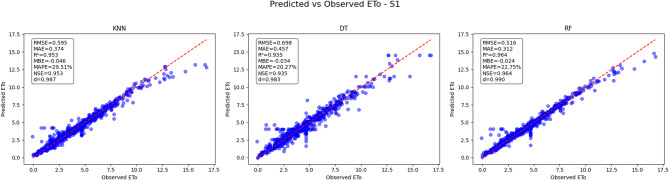
Predicted vs. Observed ETo - Scenario 1 (S1).

In Scenario 1(Fig. 23), the Random Forest (RF) model again outperforms the K-Nearest Neighbors (KNN) and Decision Tree (DT) models, with the lowest RMSE of 0.516 and MAE of 0.312, indicating better prediction accuracy. The RF model has a high R² of 0.964, showing a very strong correlation between predicted and observed ETo values. The MBE for RF is −0.024, indicating minimal bias, and the model achieves NSE of 0.964, further confirming its reliability. The KNN model shows good performance with RMSE 0.595 and MAE 0.374, while the DT model performs slightly worse with RMSE 0.698 and MAE 0.457. Both KNN and DT have slightly lower R² values (0.953 and 0.93, respectively) compared to RF. Overall, the RF model delivers the most accurate and consistent ETo predictions for Scenario 1.

#### Predicted vs. observed ETo results for scenario 2 (S2)

**Fig. 24:**
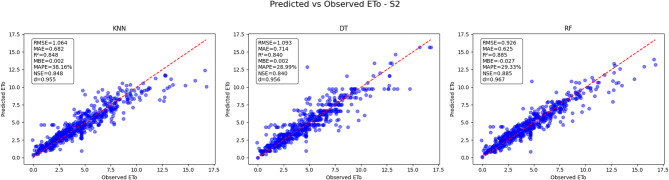
Predicted vs. Observed ETo - Scenario 2 (S2).

In Scenario 2 (Fig. 24), the RF model demonstrates superior predictive accuracy with an RMSE of 0.926, an MAE of 0.625, and an R² of 0.885, indicating a strong fit between predicted and observed ETo values. The RF model also maintains a low MBE of −0.027, suggesting minimal systematic bias, and achieves NSE of 0.885, underscoring its reliability. The KNN model exhibits slightly lower performance with an RMSE of 1.064, MAE of 0.682, and R² of 0.848, but maintains negligible bias (MBE = 0.002) and a respectable NSE of 0.860. The Decision Tree (DT) model performs moderately with an RMSE of 1.093, MAE of 0.714, and R² of 0.80, while showing a near-zero bias (MBE = 0.002) and NSE of 0.840. Visual inspection confirms that RF predictions cluster more tightly around the 1:1 line compared to KNN and DT, reflecting its enhanced precision and consistency for this scenario. Overall, Random Forest outperforms the other models in accurately estimating ETo under the conditions of Scenario 2.

#### Predicted vs. observed ETo results for scenario 3 (S3)

**Fig. 25:**
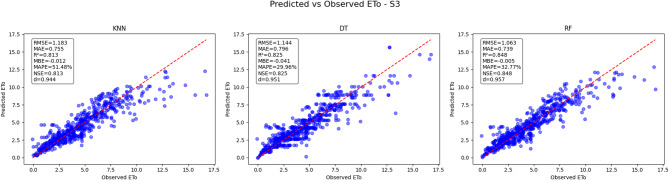
Predicted vs. Observed ETo - Scenario 3 (S3).

In Scenario 3 (Fig. 25), the RF model again demonstrates the best predictive performance, with an RMSE of 1.063 and an MAE of 0.739, indicating lower overall prediction errors compared to the other models. The RF model achieves a solid R² value of 0.848, which reflects a strong correlation between predicted and observed ETo values. Additionally, it maintains a minimal bias, with an MBE of −0.005, and an NSE of 0.848, signifying good model reliability. The KNN model shows slightly poorer performance, with a higher RMSE of 1.183 and MAE of 0.755, but with comparable R² (0.813) and a small negative bias (MBE = −0.012). The Decision Tree (DT) model performs moderately, with an RMSE of 1.144 and MAE of 0.796, accompanied by an R² of 0.825 and a slightly larger negative bias (MBE = −0.041). The RF model’s predictions align more closely with the 1:1 reference line, indicating more consistent and accurate ETo estimation for this scenario. Overall, Random Forest continues to be the most robust model in predicting ETo under Scenario 3 conditions.

#### Predicted vs. observed ETo results for scenario 4 (S4)

**Fig. 26:**
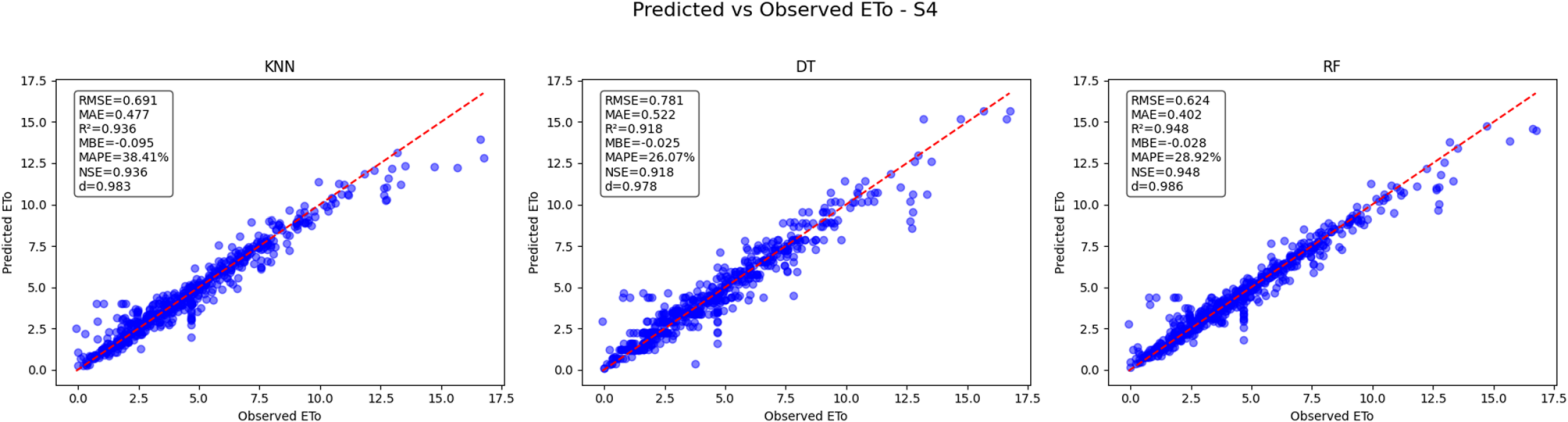
Predicted vs. Observed ETo - Scenario 4 (S4).

For Scenario 4 (Fig. 26), the RF model again exhibits superior performance with an RMSE of 0.624 and an MAE of 0.402, indicating relatively low prediction errors. It achieves the highest R² value of 0.948, reflecting a strong agreement between predicted and observed ETo values. The RF model shows minimal bias with an MBE of −0.028 and maintains an NSE of 0.948, which suggests robust predictive accuracy and reliability. DT model performs moderately well, with an RMSE of 0.781 and MAE of 0.522, a slightly lower R² of 0.918, and a small negative bias (MBE = −0.025). KNN model shows the highest RMSE and MAE values among the three models at 0.691 and 0.477 respectively, with an R² of 0.936, indicating slightly less accurate predictions compared to RF and DT. The RF model’s predictions align most closely with the 1:1 reference line, confirming its reliability and accuracy in predicting ETo for this scenario.

#### Predicted vs. observed ETo results for scenario 5 (S5)

**Fig. 27:**
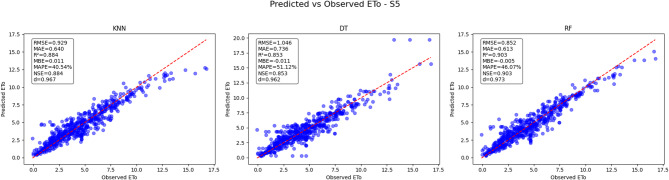
Predicted vs. Observed ETo - Scenario 5 (S5).

In Scenario 5 (Fig. 27), the RF model again demonstrates the best performance among the three algorithms, achieving an RMSE of 0.852 and an MAE of 0.613, which are lower than those of KNN and DT models. The RF model has a high coefficient of determination (R²) of 0.930, indicating a strong correlation between predicted and observed ETo values. The model’s bias is minimal, with an MBE of −0.005, and it maintains a high NSE of 0.903, highlighting its accuracy and reliability. The DT model shows moderate performance with RMSE and MAE values of 1.046 and 0.736, respectively, and an R² of 0.853. The KNN model shows an RMSE of 0.929 and an MAE of 0.640, with an R² of 0.884, performing slightly better than DT but worse than RF. Overall, the RF model consistently provides the most accurate and precise predictions for ETo across this scenario.

## Discussion

This study aimed to evaluate the predictive performance of three machine learning models K-Nearest Neighbors (KNN), Decision Tree (DT), and Random Forest (RF) in estimating reference evapotranspiration (ETrs) based on meteorological variables. The results underscore the superiority of Random Forest in capturing the non-linear relationships between meteorological inputs and ETrs, highlighting its robustness and predictive accuracy.

### Temporal dependency of meteorological variables

The temporal analysis revealed significant seasonal autocorrelation in temperature variables (Tx and Tn), consistent with previous studies that have noted the strong seasonal patterns in temperature dynamics. Temperature typically exhibits long-term dependencies across multiple lags, as observed in this study, which aligns with findings by^29^. Furthermore, while temperature variations are often gradual, other meteorological variables such as precipitation and wind speed can exhibit more erratic short-term fluctuations, impacting their temporal dependencies^30^. Solar radiation (Rs) also exhibited clear seasonal dependencies, with significant short-term correlations, in agreement with studies by ^31^.The robust nature of the Random Forest model, as demonstrated in this study, stems from its ability to effectively handle such diverse temporal dependencies and non-linear relationships present in meteorological datasets^32^. Conversely, wind speed (U2) showed weak temporal dependence, which is consistent with earlier research by^33^.This weak dependency suggests that wind speed may have a less predictable temporal pattern compared to other meteorological variables, posing challenges for models that rely heavily on sequential data.

### Correlation analysis

Correlation analysis confirmed that solar radiation, maximum temperature, and minimum temperature are the primary drivers of ETrs, with all three showing strong positive correlations with evapotranspiration. These results are consistent with studies such as those by ^30^.The robust relationships observed between these variables and ETrs emphasize their critical importance in hydrological modeling and agricultural water management^31^. Conversely, relative humidity and wind speed exhibited weaker or inverse correlations, indicating their lesser influence on ETrs compared to temperature and solar radiation^34^.This suggests that while temperature and solar radiation are direct energetic drivers of the evaporative process, humidity and wind speed play more nuanced roles, often modulating the atmospheric demand for water vapor rather than initiating the process itself^15^.Wind speed, exhibiting a moderate positive correlation, aligns with the findings of previous studies that demonstrated Random Forest’s effectiveness in capturing such relationships^35^. In contrast, relative humidity variables (HRx and HRn) showed negative correlations with ETrs, which is in agreement with findings from multiple studies, including those by Barzegar et al. and Ferreira et al., who reported similar negative impacts of relative humidity on ETo in machine-learning models^36^. Furthermore, variable-importance analysis reveals that solar radiation dominates the influence on ETr, whereas wind speed contributes the least, echoing the correlation trends identified herein^37^.

### Model performance

In terms of model performance, Random Forest outperformed both KNN and DT across all evaluation metrics (RMSE, MAE, R², MBE, KGE, NSE), indicating its superior ability to capture the complex, non-linear relationships inherent in the data. This finding is consistent with the results of previous studies that reported Random Forest achieving higher accuracy than alternative machine-learning approaches for ET₀ estimation^35,37^. Moreover, when the full suite of meteorological predictors was utilized, Random Forest consistently yielded the lowest RMSE and MAE across all input configurations, while KNN’s superiority was confined to limited-temperature inputs^37^. KNN, while effective in simpler scenarios, struggled as the feature set grew more complex, reflecting its sensitivity to the dimensionality of the data. This result is in line with the work of previous work demonstrated that KNN maintains high accuracy only with limited temperature inputs, and its performance degrades as additional meteorological variables are incorporated^38^. Decision Tree, on the other hand, demonstrated lower overall performance, particularly in handling higher-dimensional data, which aligns with the conclusions drawn by those investigations that reported Decision Trees underperform in high-dimensional ET₀ estimation scenarios^37^. These findings suggest that ensemble-based approaches, which integrate the strengths of multiple learners, may further improve ET₀ estimation accuracy, particularly when only limited meteorological data are available.

### Feature scenario evaluation

Performance varied across different feature scenarios, with Random Forest exhibiting consistent superiority in all configurations. When all meteorological variables were included, RF minimized prediction errors and achieved the highest model fit. This result corroborates the findings of previous research demonstrating Random Forest’s superior performance over alternative algorithms in evapotranspiration estimation tasks^38^. Similar superiority of Random Forest has been reported in other studies where it outperformed alternative models across diverse meteorological input combinations^35,37^. KNN performed well in simpler feature scenarios, but its accuracy declined as more predictors were incorporated, highlighting its vulnerability to the curse of dimensionality. A similar pattern was observed by other studies where an increase in the number of features led to a decrease in the reliability of distance measures, which are fundamental to KNN’s operation^15,39^. Decision Tree showed moderate performance in less complex scenarios but was less effective as the feature set expanded, with higher RMSE and MAE values, supporting the findings of previous research indicating that decision trees can struggle with high-dimensional data by creating overly complex and potentially overfitting models^15^.

### Prediction of ETrs

The results from the ETrs prediction experiments reinforced the dominance of Random Forest in terms of both accuracy and reliability. RF demonstrated minimal error and strong model fit, with high values for Nash-Sutcliffe Efficiency (NSE) and KGE, indicating excellent predictive capability. This aligns with the work of Elzain et al. and Uddin et al., who similarly utilized RF algorithms for feature selection in environmental hydrological applications due to its robustness in handling small datasets and ability to capture non-linear relationships^32^. RF achieved high NSE values in predicting ETrs. In contrast, KNN exhibited greater variability in its predictions, as noted by ^15^ and outperformed other sophisticated machine learning models like Multilayer Perceptron and AdaBoost in specific input combinations, particularly when relying solely on soil data. Decision Tree consistently underperformed, particularly in more complex feature combinations, which concurs with the observations made by^40,41^.

### Model robustness and stability

Random Forest demonstrated notable robustness and stability across varying feature scenarios, producing consistent results with lower variability in predictions compared to KNN and DT. This stability makes RF a reliable model for operational applications, especially in contexts where model generalizability is crucial. These results are consistent with the work of Elzain et al. and Uddin et al., who also highlight the efficacy of RF algorithms in environmental modeling due to their inherent robustness and capacity for handling complex, non-linear relationships within datasets^32^. Conversely, KNN and DT exhibited greater sensitivity to the chosen feature set, leading to higher variability in their predictions and suggesting that they may not perform as reliably under changing conditions. Similar conclusions were drawn by previous studies have reported analogous sensitivity of KNN and DT to feature selection, underscoring the necessity for robust feature engineering^41^. Employing ensemble-based feature selection techniques has been shown to markedly increase the stability of selected subsets, thereby mitigating the variability observed in KNN and DT models^42,43^.

### Implications for predictive modeling of ETrs

These findings have significant implications for the use of machine learning in evapotranspiration modeling. Random Forest emerged as the most suitable model for predicting ETrs, due to its ability to effectively handle complex, high-dimensional data and capture intricate variable interactions. These results align with the broader body of literature, including the works of Breiman’s seminal introduction of Random Forests and subsequent applications to soil moisture and hydrological modeling (e.g., Pan et al. demonstrated that integrating LSTM-based models with remote sensing datasets such as SMAP and ERA5 can substantially enhance ETrs prediction accuracy in heterogeneous agricultural landscapes^44^. ETr prediction Consequently, practitioners should prioritize hyper-parameter optimization of Random Forests to fully exploit their capacity for capturing nonlinear ET dynamics across diverse climatic regimes^31,35^.These findings have significant implications for the use of machine learning in evapotranspiration modeling. Random Forest emerged as the most suitable model for predicting ETrs, due to its ability to effectively handle complex, high-dimensional data and capture intricate variable interactions. These results align with the broader body of literature, including the works of the study by (e.g., Zhao et al. demonstrated comparable performance of XGBoost over Random Forest in daily ETref estimation^45^. ^37^ recommended Random Forest as the model of choice for environmental prediction tasks. Consequently, future studies should prioritize hyper-parameter optimization and ensemble diversity to further leverage Random Forest’s robustness across heterogeneous agro-ecological settings. KNN, while useful for simpler datasets, proved less effective as the complexity of the input features increased, supporting the observations of as demonstrated in earlier comparative studies of evapotranspiration estimation, where Random Forest consistently outperformed KNN in high-dimensional settings^46–48^.

### Limitations and future research

This study has several limitations. The models were trained on historical meteorological data, which may limit their applicability to real-time or future datasets that exhibit different temporal or spatial patterns. These concerns were also raised by other research, which suggests that AI-based methods may produce smoother forecast results, potentially underestimating the magnitude of extreme weather events^49^. Additionally, while Random Forest provided accurate predictions, its black-box nature limits interpretability, an aspect that could be addressed in future work by exploring more transparent models or hybrid approaches that combine predictive power with interpretability. Further research could also incorporate spatial variability in meteorological data, as topographical and land use factors could influence evapotranspiration rates. Hybrid models combining machine learning and time-series analysis, such as Random Forest with ARIMA or Long Short-Term Memory (LSTM) networks, could be explored to capture both temporal and spatial dynamics of ETrs more effectively, as suggested by earlier studies^50^. Furthermore, the reliance on global potential evapotranspiration products without site-specific ground validation represents another limitation, particularly in underrepresented regions where the generalizability of findings may be constrained^51^.

### Limitations and future work

One limitation is that the dataset is region-specific, potentially restricting the generalizability of the models to other climatic zones. Additionally, the study focused on standard hyperparameter tuning; advanced optimization techniques (e.g., Bayesian optimization) may yield further improvements.

Future research directions include expanding the geographic scope, incorporating additional climate variables (e.g., soil moisture), and applying more sophisticated machine learning techniques, such as deep learning, to enhance ETo prediction accuracy. Furthermore, exploring innovative feature engineering methods and model calibration approaches could address the limitations of existing ETo models and improve their applicability in diverse environmental settings.

## Conclusion

This study provides a comprehensive evaluation of machine learning approaches for estimating daily reference evapotranspiration (ETo) using meteorological inputs. Temporal dependency analysis revealed that while variables such as temperature, solar radiation, and ETo itself exhibit strong seasonal patterns, their short-term temporal dependencies are limited. Conversely, wind speed and relative humidity show near-random temporal behavior. These findings justify the use of static, non-sequential models for ETo prediction, simplifying the modeling framework without significant loss of accuracy.

Among the evaluated algorithms, Random Forest consistently outperformed K-Nearest Neighbors and Decision Tree models in terms of predictive accuracy, robustness, and generalizability across varying parameter settings and feature sets. Its ability to capture complex, nonlinear relationships between meteorological variables and ETo underpins its superior performance. This confirms Random Forest as a reliable and practical tool for operational hydrological applications, irrigation management, and climate-adaptive agriculture.

Notably, this work emphasizes that detailed time-series modeling may offer limited additional benefit when daily meteorological inputs are available, which is an important consideration for model design in resource-constrained contexts. However, the study’s scope is limited to daily-scale data and specific climatic conditions; thus, future research should investigate the model’s transferability across diverse climatic regions and explore integration with spatial datasets to further enhance prediction accuracy.

Overall, this study contributes a clear framework for selecting appropriate machine learning techniques for ETo estimation, balancing complexity and performance. It highlights the value of leveraging data-driven approaches that align with the intrinsic temporal characteristics of meteorological drivers, offering a practical path forward for improving water resource management under changing environmental conditions.

## Data Availability

Data is contained within the article.
